# A rapid and visual turn-off sensor for detecting copper (II) ion based on DNAzyme coupled with HCR-based HRP concatemers

**DOI:** 10.1038/srep43362

**Published:** 2017-03-07

**Authors:** Wentao Xu, Jingjing Tian, Yunbo Luo, Longjiao Zhu, Kunlun Huang

**Affiliations:** 1 Beijing Advanced Innovation Center for Food Nutrition and Human Health, College of Food Science & Nutritional Engineering, China Agricultural University, Beijing 100083, China; 2 Beijing Laboratory for Food Quality and Safety, College of Food Science and Nutritional Engineering, China Agricultural University, Beijing 100083, China

## Abstract

To solve the requirement of on-site, rapid, and visual detection of copper (II) (Cu^2+^) in aqueous solution, a turn-off sensor for detecting copper (II) ion was developed based on Cu^2+^-dependent DNAzyme as the recognition element and hybridization chain reaction (HCR)-based horseradish peroxidase (HRP) concatemers as the signal amplifier and the signal report element. The detection unit, which was composed of the immobilized Cu^2+^-dependent DNAzyme coupled with HCR-based HRP concatemers via Waston-Crick base pairing, could catalyze hydrogen peroxide (H_2_O_2_) via TMB, generating obvious green color and turning yellow after sulfuric acid termination with optical absorption at 450 nm. Upon Cu^2+^ addition, the substrate strand of the Cu^2+^-dependent DNAzyme concatenated with the HCR-based HRP complex was irreversibly cleaved, efficiently causing dramatic reduction of the detection signal. Under optimal conditions, the detection signal decreased with the concentration of Cu^2+^ in 5 min, exhibiting a linear calibration from 0.05 to 3 μM with a detection limit of 8 nM. The sensor also displayed a high selectivity for Cu^2+^ given the specificity and anti-interference of the detection unit, and this system was applicable for monitoring Cu^2+^ in real water samples. Generally speaking, the proposed sensor exhibits good potential in environment surveys.

Heavy metal detection has received much attention in scientific research due to their serious threat to the environment and health^1^,^2^,^3^,^4^. Of particular interest there has been the detection of copper (II) ion, a dangerous pollutant that causes adverse afflictions in people upon excess intake, including liver or kidney damage, gastrointestinal disturbance^5^, Parkinson’s disease, Wilson’s disease and Alzheimer’s disease^3^,^4^. Considering its toxicity, the Chinese Ministry of Health (CMH), U.S. Environment Protection Agency (EPA) and World Health Organization (WHO) regulate maximum permissible levels of copper (II) of less than 15 μM^6^, 20 μM^7^ and 31 μM in water^8^, respectively. Thus, instrumental analytical techniques for Cu^2+^ detection have been developed, such as inductively coupled plasma mass spectrometry (ICP-MS)^9^, atomic absorption spectrometry (AAS)^10^, inductively coupled plasma atomic emission spectrometry (ICP-OES) and fluorescence spectrometry^11^,^12^. However, these widely used instrumental techniques are rather complicated, requiring sophisticated apparatus and professional personnel, which are obstructive to further application. To keep pace with expectations in future on-site rapid testing, the development of new sensing techniques for Cu^2+^ is a hot issue in analytical chemistry in recent years.

Among numerous new sensors, DNAzyme-based biosensors are of particular interest^13^,^14^. DNAzyme, a type of functional nucleic acid, is able to catalyze a series of reactions. Recently, two types of DNAzyme have been a hot topic of research. One type of DNAzyme is G-quadruplex-based DNAzyme, which possesses mimic-horse radish peroxidase (HRP) activity to catalyze specific substrate for chromogenic reaction^15^,^16^,^17^,^18^,^19^. The other type is metal ion-dependent DNAzyme, Which could depend on specific metal ions as cofactors, such as Ag^+^ ions^20^, Na^+^ ions^21^,^22^, Cu^2+^ ions^23^,^24^,^25^, Pb^2+^ ions^26^,^27^, Mg^2+^ ions^28^, Zn^2+^ ions^29^,^30^, Ca^2+^ ions^31^,^32^, Cd^2+^ ions^33^, UO_2_^2+^ ions, Hg^2+^ ions^34^,^35^, and lanthanide ions^36^,^37^,^38^. Most metal ions activate specific DNAzyme via cleavage. According to the nucleotide type of cleavage site (ribonucleotide and deoxyribonucleotide), metal ion-dependent DNAzyme classified as RNA-cleaving DNAzyme and DNA-cleaving DNAzyme. Cu^2+^-specific DNAzyme, which belongs to DNA-cleaving DNAzyme with the characteristics of easy synthesis, lower cost and better stability, leads to site-specific cleavage at the guanine position of the substrate in catalytic/substrate multiple strands, which makes it an ideal element for Cu^2+^ ion detection due to inherent advantages of outstanding stability under harsh conditions and high selectivity comparable to antibodies^14^,^39^,^40^. Over the past decade, different DNAzyme-based biosensors for Cu^2+^ using various signal transduction methods have been developed, including fluorescence^41^,^42^, colorimetry^39^,^42^,^43^,^44^ and lateral flow nucleic acid techniques^45^. Such DNAzyme-based sensing strategies require simpler apparatus and less professional personnel than traditional techniques.

Interestingly, hybridization chain reactions (HCR), which generate linear DNA nanostructures arisen by self-assembly of short DNA fragments through a cross-opening process, have recently become one of the most active research areas in molecular biology and DNA diagnostics relying on several unique advantages^46^,^47^. Firstly, signal amplification via Watson-Crick base pairing is easier to achieve, which can lead to ultrasensitive detections. Secondly, HCR exhibit the potential of signal reporting via special labels, which pave the way toward multi-target sensing platforms. However, HCR rely on reaction time and are temperature-dependent, so a relatively long time or appropriate temperature likely triggers false positives that interfere with the results. In our research, the HCR was prepared in advance via ingenious hairpin design, effectively eliminating undesirable initiation. Herein, we achieved the proof-of-concept to integrate Cu^2+^-dependent DNAzyme with HCR-based HRP concatemers for rapid detection of Cu^2+^ semi-quantitatively observed by the naked eye and quantitatively monitored by a portable spectrophotometer in 5 min, exhibiting immense potential for on-site detection.

## Experiment Section

### Reagents and Apparatus

All oligonucleotides were HPLC-purified and synthesized at Invitrogen (Life Technologies, Beijing, China). The substrate strand and catalytic strand for copper (II)–dependent DNAzyme were designed according to the literature^42^. The sequences of the oligonucleotides are as follows:

Substrate strand for Cu^2+^-dependent DNAzyme (S_1_):

5′-GGGGGGGGGGGGGGGGGGGGGGTTTTTTTAGCTTCTTTCTAATACGGCTTACCTTTTTTTTTT-Biotin-3′

Catalytic strand for Cu^2+^-dependent DNAzyme (S_2_):

5′-GGTAAGCCTGGGCCTCTTTCTTTTTAAGAAAGAAC-3′

Linker-labeled initiator (S_3_):

5′-CTGAGCTTCGGATTCTGTTTGGCCCCCCCCCCCCCCCCCCCCCC-3′

Hairpin DNA (H_1_):

5′-Biotin-GGCCAAACAGAATCCGAAGCTCAGACCCTGCTGAGCTTCGGATTCTGT-3′

Hairpin DNA (H_2_):

5′-Biotin-CTGAGCTTCGGATTCTGTTTGGCCACAGAATCCGAAGCTCAGCAGGGT-3′

The ingenious design of hairpins should meet the following requirements: Hairpins (H_1_ and H_2_) should possess the same number of bases and the same number of sticky ends. Noticeably, the sticky ends of H_1_ locates at the 5′-end while that of H_2_ locates at the 3′-end. To better promote HCR, nearly half fragment of H_2_ at the 5′-end was truncated as the initiator strand. High binding polystyrene 96-welll microplate was purchased from Costar (NY, USA). In addition, 4-(2-hydroxyethyl) piperazine-1-ethanesulfonic acid (HEPES), ascorbate, streptavidin (SA), bovine serum albumin (BSA), and streptavidin-horseradish peroxidase (SA-HRP) were obtained from Sigma-Aldrich Chemical Co. (St. Louis, MO, USA). Tetramethylbenzidine (TMB) coloring solution was purchased from Biyuntian Co. Ltd (Beijing, China). Ultrapure water was used throughout the experiment and was obtained from a Milli-Q water purifying system (resistivity ≥18.2 MΩ cm^−1^, Milli-Q, Millipore). Other chemicals were of analytical grade and were used without further purification.

Gel electrophoresis was conducted on a Molecular Imager Gel Doc XR (Bio-Rad, Canada). Visual measurements were carried out with a portable spectrophotometer (NS810, Shenzhen 3nh Technology Co., Ltd). The transmission electron micrograph (TEM) was performed on a JEOL JEM-1400 Transmission electron micrograph (Germany).

### Immobilization of copper (II)–dependent DNAzyme

To immobilize Cu^2+^-dependent DNAzyme, 2 μg/mL SA (100 μL) in carbonate buffer solution (CBS, 15 mM Na_2_CO_3_, 35 mM NaHCO_3_, pH 9.6) was added to wells and incubated at 37 °C for 2 h. After washing three times with 250 μL wash buffer (0.15 M NaCl, 10 mM K_2_HPO_4_, 1 mM NaH_2_PO_4_, 0.05% Tween 20, pH 7.4), the wells were blocked with 200 μL of 1% BSA per well at 37 °C for 2 h. Then, 100 μL biotinylated S_1_ (120 nM) dissolved in phosphate buffer solution (PBS, 0.15 M NaCl, 10 mM K_2_HPO_4_, 1 mM NaH_2_PO_4_, pH 7.4) was added to each well of 96-well plates and incubated at 37 °C for 1 h. Next, the wells were washed three times with wash buffer, incubated with S_2_ (150 nM) dissolved in 50 mM HEPES buffer (pH 7.0) containing 1.5 M NaCl at 37 °C for 100 min and washed three times again.

### Preparation of HCR-based HRP concatemers

Biotinylated hairpins (H_1_ and H_2_) were heated at 95 °C for 5 min and then cooled to 4 °C slowly for 1.5 h before use. The hybridization chain reaction (HCR) proceeded at 37 °C for 1 h, composed of 200 nM S_3_, 1 μM H_1_ and 1 μM H_2_ in Tris-HCl Buffer (10 mM Tris, 500 mM NaCl, 1 mM MgCl_2_, pH 7.4). Afterwards, SA-HRP (500 ng/mL) was added to the HCR system and incubated at 37 °C for 1 h to form the HCR-HRP complex. HCR products were detected by 2% EB-stained agarose gel electrophoresis. HCR-based HRP concatemers was inspected by the transmission electron micrograph^48^.

### Assembly of the detection unit and copper (II) assay

Immobilized Cu^2+^-dependent DNAzyme was concatenated with HCR-based HRP complex (100 μL) in each well at 37 °C for 2 h. After washing three times, the detection unit of Cu^2+^ was achieved. Then a fixed concentration of Cu^2+^ (CuSO_4_, 100 μL) dispersed in the reaction buffer (1.5 M NaCl, 50 mM HEPES and 50 μM ascorbate) was incubated with the Cu^2+^-detection unit in each well at 37 °C for 5 min. The same volume of reaction buffer was used as the negative control. After washing three times, 100 μL TMB coloring solution was added to each well. Then, the samples were incubated at room temperature away from light for 5 min, and 50 μL H_2_SO_4_ (2 M) was added to terminate the chromogenic reaction. The absorbance values at 450 nm (OD_450_) were monitored by a portable spectrophotometer.

### HPLC-DAD analysis

Conditions for DNA analysis followed the description by Wilde *et al*. with some modifications^49^. HPLC analyses were performed on an Agilent 1100 HPLC system together with a diode array detector (DAD). A TSKgel DNA-NPR (4.6 mm i.d. × 7.5 cm, Tosoh, Japan) was employed. DNA samples were separated by buffer A (20 mM Tris, pH 9.0) and buffer B (1 M NaCl in 20 mM Tris, pH 9.0) according to the following gradient: 25–45% B in 0–0.13 min, 45–50% B in 0.13–4 min, 50–64% B in 4–25 min, 25% B in 25.1–35 min. The flow rate was 0.4 mL/min with an injection volume of 10 μL. DNA elution was monitored by UV absorbance at 260 nm. All chromatography was performed at 35 °C.

### Detection of real water samples

Non-contaminated commercial bottled purified water was purchased from the local supermarket. Lake water was collected from Beijing Olympic Green Park, Beijing, China. Domestic sewage was obtained from the Beijing north river sewage treatment factory, Chaoyang District, Beijing, China. Firstly, the water samples were gently mixed with Cu^2+^ standard solution, generating two final Cu^2+^ concentrations (0.5 μM and 2.5 μM). Then, a 90-μL water sample was mixed with 10 μL 10× reaction buffer (15 M NaCl, 500 mM HEPES and 500 μM ascorbate), and the obtained 100-μL solution was treated using the same protocol as noted above for the Cu^2+^ detection. The ICP-MS analysis for Cu^2+^ in real water samples was based on Szpunar’s study^6^.

## Result and Discussion

### Design principle of turn-off sensor

**Figure 1:**
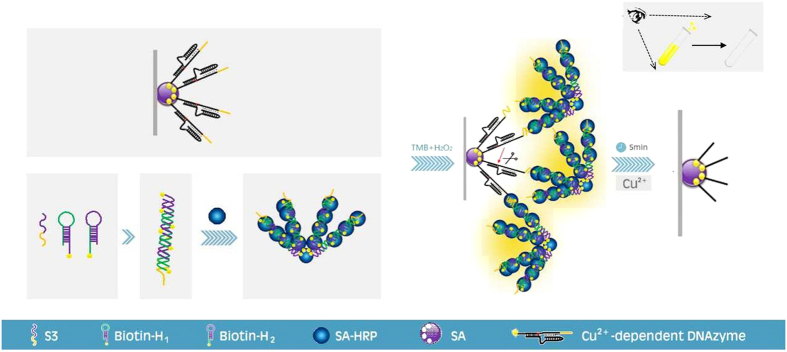
Schematic of the design of the turn-off sensor for the detection of copper (II) ion (Cu^2+^) based on Cu^2+^-dependent DNAzyme coupled with HCR-based HRP concatemers.

The rapid and visual DNA concatenation sensor for Cu^2+^ detection is schematically illustrated in Fig. 1. Beforehand, SA was immobilized onto the high binding polystyrene well via hydrophobic effect. Then, S_1_ was captured via biotin-streptavidin combination, which was flanked with spacer sequences of poly-thymine (poly-T) oligonucleotides at the 3′ end and with linker sequence of poly-guanine (poly-G) oligonucleotides at the 5′ end. Via Watson-Crick base pairing at the 3′ terminus and the formation of the DNA triplex at the 5′ terminus, S_2_ coupling with S_1_ formed the Cu^2+^-dependent DNAzyme. So Cu^2+^-dependent DNAzyme was coated onto the well via biotin-streptavidin linkage to identify the target.

Simultaneously, SA-HRP and the nicked DNA double-helix formed a complex. In the presence of linker-labeled initiator, biotinylated hairpin 1 (H_1_) first underwent an unbiased strand-displacement reaction to open the hairpin from the 5′ sticky end. The newly exposed sticky nucleates of H_1_ opened biotinylated hairpin 2 (H_2_) and underwent a strand-displacement reaction, exposing another sticky end. Similarly, a chain reaction of hybridization events was propagated to form the biotinylated nicked DNA double-helix, acting as the signal amplifier. In the presence of SA-HRP, which often served as the signal output element, the linker-labeled HCR-based HRP complex formed via biotin-streptavidin linkage. Given that HCR-based HRP complex not only amplified detection signal but also catalyzed hydrogen peroxide (H_2_O_2_) via TMB, generating obvious yellow color visible to the naked eye and optical intensity at 450 nm (OD_450_) after sulfuric acid termination, the HCR-based HRP complex acted as a signal amplification element as well as a signal report element.

Finally, Cu^2+^-dependent DNAzyme concatenated with the HCR-based HRP complex was achieved via Watson-Crick base pairing. Upon target Cu^2+^ introduction, S_1_ of the Cu^2+^-dependent DNAzyme concatenated with the HCR-based HRP complex was irreversibly cleaved. We hypothesized that the cleaved pieces were released and totally washed, leading to the dramatic reduction in OD_450_ values and absence of yellow color.

### Optimization of the immobilized Cu^2+^-dependent DNAzyme

**Figure 2:**
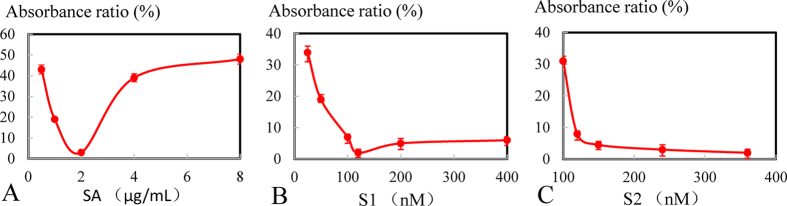
Optimization of immobilized Cu^2+^-dependent DNAzyme. (**A**) Absorbance ratio (%) of the turn-off sensor with different concentrations of SA (μg/mL). The concentrations from the left are 0.5, 1, 2, 3, and 8 μg/mL. (**B**) Absorbance ratio (%) of the turn-off sensor with different concentrations of S_1_ (nM). The concentrations from the left are 25, 50, 100, 120, 200, and 400 nM. (**C**) Absorbance ratio (%) of the turn-off sensor with different concentrations of S_2_ (nM). The concentrations from the left are 100, 120, 150, 240, and 360 nM. Error bars represent standard deviation (N = 3).

Given that the Cu^2+^-dependent DNAzyme was immobilized onto the surface via biotin-streptavidin linkage, the concentrations of SA, biotinylated substrate strand (S_1_) and Cu^2+^ catalytic strand (S_2_) were optimized. As illustrated in Fig. 2, the following equation was employed: absorbance ratio (%) = A_0_/A × 100 at 450 nm^7^, where A_0_ and A represent the optical intensity after and before Cu^2+^ sample addition, respectively. Smaller the ratio, more remarkable the signal variation was, indicating the more appropriate detection conditions. In Fig. 2A, with the SA concentration ranging from 0.5 to 8 μg/mL, the absorbance ratio (%) exhibited a wave trough and a valley point at 2 μg/mL of SA, presumably due to stereo-hindrance effects. Small amounts of SA could not capture sufficient amounts of the biotinylated substrate strand, whereas excess SA would lead to steric hindrance, reducing binding with the strand^50^. In Fig. 2B, the ratio gradually declined until reaching a plateau at 120 nM from low dose to high dose, mainly because the combination of streptavidin with biotin conformed to the molar ratio of one to four (SA: 2 μg/mL ≈ 30 nM)^51^,^52^. In Fig. 2C, the ratio was reduced from 100 to 150 nM and subsequently maintained a relatively low value, indicating that slightly excessive S_2_ compared with S_1_ was ideal for saving experimental materials. In general, 2 μg/mL of SA, 120 nM of S_1_ and 150 nM of S_2_ were optimal.

### Characterization of the Chromatographic Behavior of the Cu^2+^-dependent DNAzyme on a DNA-NPR Column

**Figure 3:**
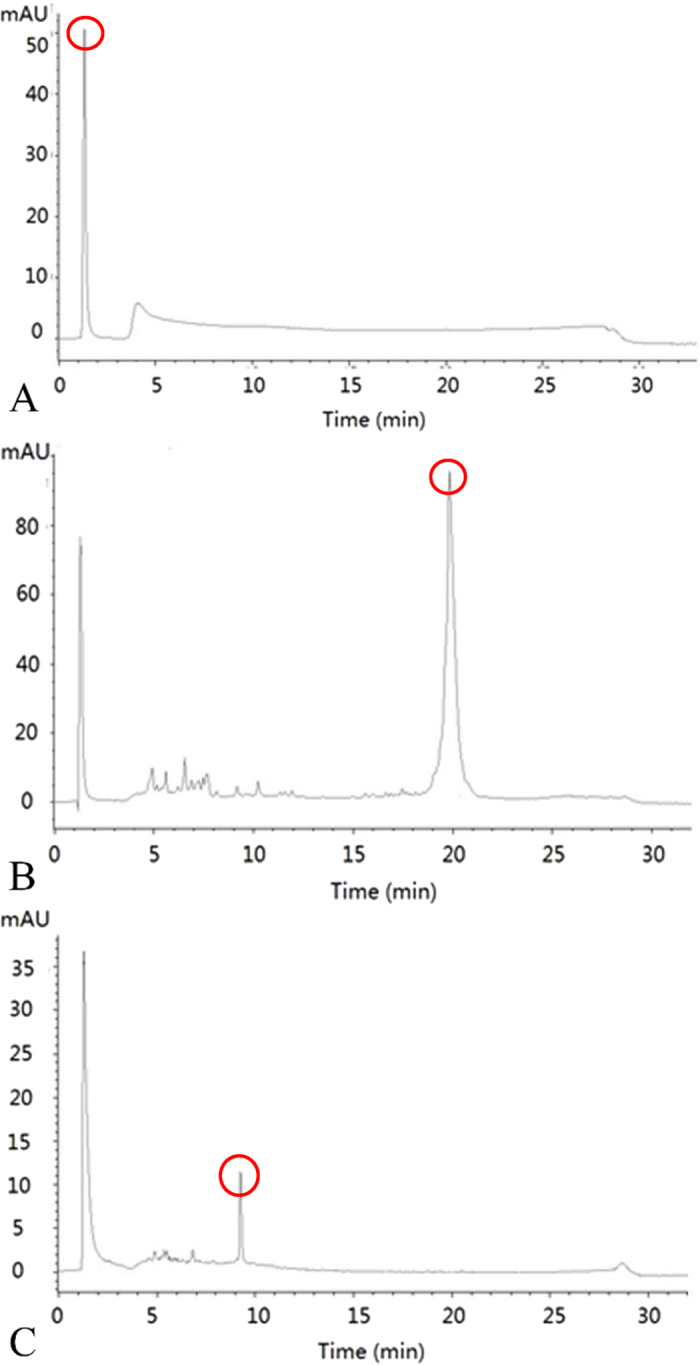
Characterization of Chromatographic Behavior of Cu^2+^-dependent DNAzyme on a DNA-NPR Column. (**A**) Characterization of chromatographic behavior for the negative control of the reaction buffer on a DNA-NPR Column. (**B**) Characterization of chromatographic behavior for the non-dissected Cu^2+^-dependent DNAzyme on a DNA-NPR Column. (**C**) Characterization of chromatographic behavior for Cu^2+^ (1 mM) cleavage of the Cu^2+^-dependent DNAzyme on a DNA-NPR Column.

Anion exchange chromatography is a widely used technique for separating negatively-charged DNA molecules. The TSK gel DNA-NPR column is a polymer-based anion exchange column for improved DNA resolution over a wide molecular weight range. The capability of the TSK gel DNA-NPR column to separate both high and low molecular weight DNA fragments has been demonstrated by Wilde *et al*.^49^. Figure 3A,B and C present typical elution profiles of the negative control of the reaction buffer, before and after the Cu^2+^ cleavage of the Cu^2+^-dependent DNAzyme, respectively. In Fig. 3A, the negative control showed a high response value at the retention time of 1.187 min with the corresponding peak area of 917. Compared with the negative control, Fig. 3B revealed a relatively high response value at the retention time of 19.835 min with the corresponding peak area of 3073, indicating that the chromatographic peak resulted from the non-dissected Cu^2+^-dependent DNAzyme. In Fig. 3C, there was no response at 19.835 min, whereas a new high chromatographic peak appeared at the retention time of 9.239 min compared with Fig. 3B, suggesting the split Cu^2+^-dependent DNAzyme. Based on these scientific data, we concluded that the Cu^2+^-dependent DNAzyme was cut into small pieces after Cu^2+^ sample addition.

### Optimization of HCR-based HRP concatemers

**Figure 4:**
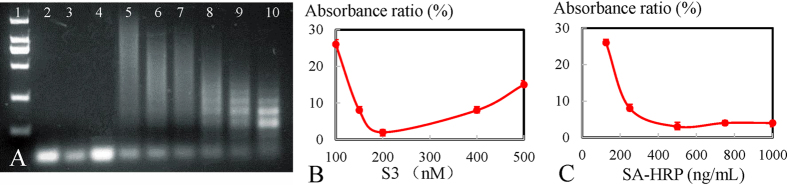
Optimization of HCR-based HRP concatemers. (**A**) EB-stained agarose gel electrophoresis demonstrated S_3_-initiated HCR: lane 1, 2000-bp marker from top to bottom was 2000, 1000, 750, 500, 250, and 100 bp; Lane 2, 1.0 μM H_1_; Lane 3, 1.0 μM H_2_; Lane 4, mixture of 1.0 μM H_1_ and H_2_; Lanes 5–10, the presence of S_3_ (100 nM, 150 nM, 200 nM, 400 nM, 500 nM, and 600 nM) with a mixture of H_1_ and H_2_. HCR time: 1 h. (**B**) Absorbance ratio (%) of the turn-off sensor with different concentrations of S_3_ (nM). The concentrations from the left are 100, 150, 200, 400, and 500 nM. (**C**) Absorbance ratio (%) of the turn-off sensor with different concentrations of SA-HRP (ng/mL). The concentrations from the left are 125, 250, 500, 750, and 1000 ng/mL. Error bars represent standard deviation (N = 3).

As the signal amplifier and signal report element, HCR-based HRP concatemers were optimized, including HCR and the dosage of SA-HRP. To the best of our knowledge, the signal amplification effect of HCR depends on the length of the hybridization chain. Longer the HCR product, the better the effect of signal amplification is. Inspiringly, the longitude of the hybridization chain was hinged on the ratio between the dose of the initiator and two closed hairpins^53^. As proof-of-principle (Fig. 4A), the HCR between the initiator (S_3_) and H_1_ and H_2_ was examined by agarose gel electrophoresis. In the absence of S_3_, a bright band of H_1_ (lane 2) and H_2_ (lane 3) could be observed, and the HCR between H_1_ and H_2_ was inhibited (lane 4). However, emission bands of high-molecular-weight structures could be observed in lanes 5–10, indicating that higher the ratio of the initiator to hairpins, lower the molecular weight of the HCR product was. A 1:10 ratio of initiator to hairpins triggered the longest hybridization chain, reaching a length of 2 kb. Ratios of 1.5:10 and 2:10 are similar, triggering a longer length of products, which is greater than 1.5 kb and shorter than 2 kb. The 1:10 ratio, corresponding to 100 nM S_3_, 1 μM H_1_ and 1 μM H_2_ was optimal. However, the absorbance ratio at 450 nm was reduced from 100 to 200 nM of S_3_ and slightly went up at higher concentrations (Fig. 4B), indicating that 200 nM of S_3_ (corresponding to 1 μM H_1_ and H_2_) was optimal. Comprehensively, 200 nM of S_3_ was selected, considering the ideal signal amplification effect, the obvious signal report and no false positive results. Regarding the concentration of SA-HRP (Fig. 4C), the absorbance ratio at 450 nm was reduced until a stable region was achieved when the concentration of SA-HRP was denser than 500 ng/mL, demonstrating that the optimal dosage was 500 ng/mL in the absence of dissipation.

### Characterization of HCR-based HRP concatemers

**Figure 5:**
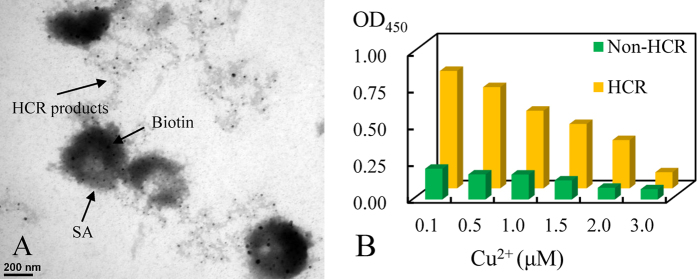
The characterization of HCR-based HRP concatemers. (**A**) Transmission electron microscope (TEM) of HCR-based HRP concatemers. (**B**) Comparison of OD_450_ with different concentrations of Cu^2+^ (μM) between with and without HCR-HRP. Experimental conditions of HCR: SA, S_1_, S_2_, S_3,_ H_1_/H_2_ and SA-HRP were 2 μg/mL, 120 nM, 120 nM, 200 nM, 1 μM/1 μM and 500 ng/mL, respectively; Experimental conditions of Non-HCR: SA, 5′, 3′-Biotin-S_1_, S_2_, and SA-HRP were 2 μg/mL, 120 nM, 120 nM and 500 ng/mL, respectively. The detection procedure was carried out as described in the Experimental section.

HCR products, serving as a typical non-enzymatic signal amplification element, were inspected by agarose gel electrophoresis as shown in Fig. 4A. The emission band demonstrate that HCR products were successfully formed. And the length of HCR products showed a S_3_ dose-response effect. When adding SA-HRP into biotinylated HCR nanostructures, an expected color change was appeared. It further confirmed that HCR-based HRP concatemers were successfully fabricated. The color change was attributed to HCR-based HRP complex could catalyze chromogen to produce chromogenic reaction. The detail color image were showed in Fig. S1. Then, transmission electron microscope (TEM) was further employed to observe the morphology of the assembled HCR-based HRP concatemers. As shown in Fig. 5A, morphology with helical concatemers was observed. The result suggested the successful formation of the HCR-based HRP concatemers. In addition, a comparison study with and without HCR-HRP have been conducted to give further validation. As shown in Fig. 5B, OD_450_ of the HCR-based system was obviously higher than that of the non-HCR system, and the sensitivity was noticeably improved, clearly indicating that the signal amplification effect of HCR process was significant, which was at least 4-fold greater than that in the non-HCR method.

### Optimization of Cu^2+^-mediated cleavage

**Figure 6:**
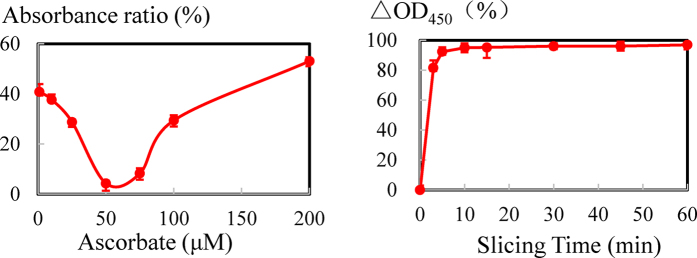
Optimization of Cu^2+^-mediated cleavage. (**A**) Absorbance ratio (%) of the turn-off sensor with different concentrations of ascorbate (μM). The ascorbate concentrations from the left are 1, 10, 25, 50, 75, 100, and 200 μM. (**B**) Absorbance ratio (%) of the turn-off sensor with the time of Cu^2+^-mediated cleavage (min). The time intervals from the left are 0, 3, 5, 10, 15, 30, 45 and 60 min. Error bars represent standard deviation (N = 3).

Referring to the literature, a suitable dosage of ascorbate could apparently promote Cu^2+^-mediated cleavage of specific DNAzyme in slicing buffer (1.5 M NaCl, 50 mM HEPES, pH 7.0)^42^. Therefore, the influence of different concentrations of ascorbate ranging from 1 to 200 μM on visual readout was studied. As shown in Fig. 6A, the absorbance ratio at 450 nm was reduced and then increased at the concentration of 50 μM ascorbate. These results indicate that concentrations of ascorbate less than 50 μM enhanced the cleavage reaction, whereas concentrations greater than 50 μM suppressed the reaction. We concluded that 50 μM ascorbate in the slicing buffer was the ideal dosage, and this finding was consistent with Liu’s results^42^.

To promote our Cu^2+^ turn-off censor for on-site rapid detection, the time of Cu^2+^-mediated cleavage was researched. As shown in Fig. 6B, the following equation was used to characterize the cleavage efficiency: ΔOD_450_ (%) = [OD_450_ (negative control) −OD_450_ (sample)]/OD_450_ (negative control) × 100%^7^. The curve of cleavage efficiency rapidly ascended, reaching a plateau after more than 5 min. Three minutes after the addition of Cu^2+^ samples, the cleavage efficiency was greater than 80%. After 5 min, the efficiency reached 92%. Given that the immobilized Cu^2+^-dependent DNAzyme coupled with HCR-based HRP concatemers was prepared on the surface in advance, the actual reaction time after Cu^2+^ sample introduction was in 5 min, indicating great promise for on-site rapid testing.

### Detection performance of the Cu^2+^ biosensor

**Figure 7:**
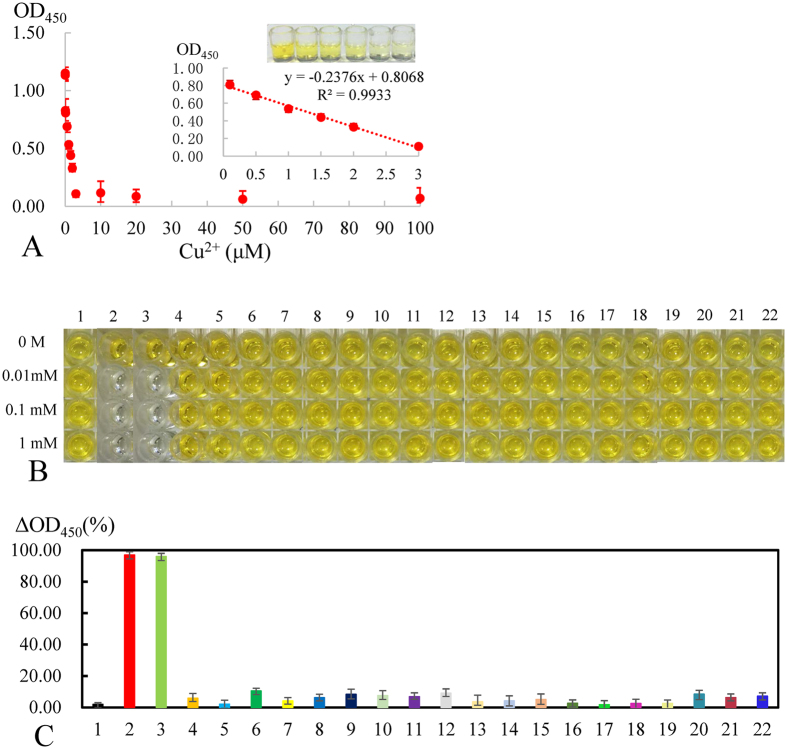
Calibration curve and selectivity of the sensor. (**A**) Calibration curve for colorimetric determinations of different concentrations of Cu^2+^ by the turn-off sensor. Inset: Calibration plot and color change of Cu^2+^ concentration vs. absorbance signal in the range from 0.05 to 3 μM. The dotted line represents the linear fit to the data. Error bars represent standard deviation (N = 3). The chromogenic results (**B**) and OD_450_ (**C**) of the Cu^2+^ turn-off sensor before and 5 min after the addition of different metal ions of definite concentrations. Error bars represent standard deviation (N = 3).

As shown in Fig. 7A, given that the Cu^2+^-dependent DNAzyme was sensitive to the target ion when the concentration of Cu^2+^ present in samples increased from 0 nM to 100 μM, the final visual readout of the Cu^2+^ turn-off sensor decreased accordingly until reaching a plateau. The calibration curve was monitored by optical density at 450 nm with corresponding Cu^2+^ concentrations within the range of 50 nM to 3 μM. The linear regression equation was OD_450_ = −0.2376 × [c (Cu^2+^)] + 0.8068 (R^2^ = 0.9933), with the detection limit (LOD) of 8 nM according to 3σrule [mean absorbance of blank+3σ (standard deviation of the blank)]. According to the regulations introduced by the Chinese Ministry of Health, US EPA and WHO, the maximum permitted levels of Cu^2+^ in drinking water are 15 μM, 20 μM and 31 μM, respectively, and this detection limit is much lower than the regulated level. Moreover, the analytical performances of different optical DNAzyme-based assays developed for Cu^2+^ analysis are summarized, and the sensitivity of this proposed method is competitive compared with these techniques (Table S1).

The specificity of the Cu^2+^ turn-off sensor was evaluated by challenging the system against other metal ions, including Co^2+^, Ti^2+^, Fe^2+^, Fe^3+^, Mn^2+^, Ca^2+^, Ni^2+^, Mg^2+^, Zn^2+^, Cd^2+^, Pb^2+^, Hg^2+^, Ba^2+^, Sr^2+^, UO_2_^2+^, Eu^3+^, Tb^2+^, Ag^+^, Al^3+^ and mixed ions. To evaluate the selectivity more accurately and more comprehensively, each metal ion mentioned above was analyzed at three different concentrations of 10 μM, 100 μM and 1 mM. As indicated in Fig. 7B and C, in contrast to the significant response of Cu^2+^ ion, other metal ions exhibited minimal interference with Cu^2+^ detection. Hence, the results exhibited high selectivity toward Cu^2+^ compared with other test metal ions given the specific function of the Cu^2+^-dependent DNAzyme.

### Determination of Cu^2+^ in water samples

**Figure 8:**
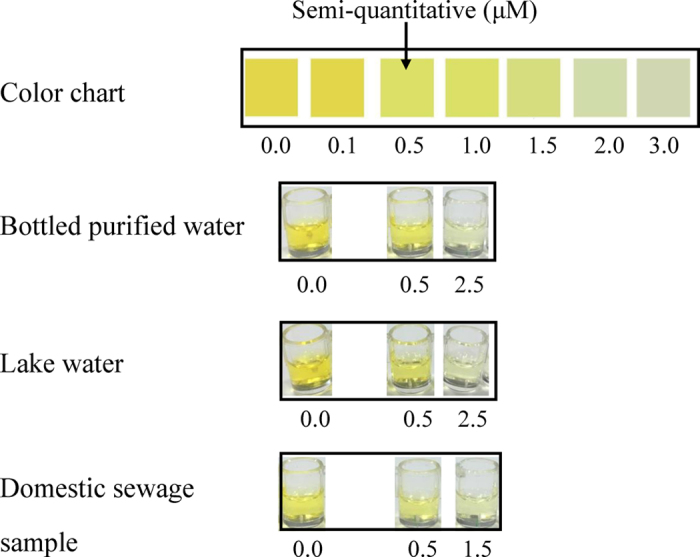
Color chart and experimental images of real sample from bottled purified water, Lake water and Domestic sewage sample.

To further elucidate the analytical reliability and applicable potential of the Cu^2+^ turn-off sensor for testing real samples, three different water samples were monitored by using our developed Cu^2+^ sensor and ICP-MS. As shown in Fig. 8, a color chart was designed to assist the qualitative analysis and semi-quantitative LOD of 0.5 μM was clearly visualized by the naked eye. 0.5 μM, 1.5 μM and 2.5 μM of Cu^2+^ could be inspected by the naked eye semi-quantitatively. Therefore, more than 0.5 μM of Cu^2+^ could be inspected by the naked eye. In addition, the detection performance by our strategy for the water samples spiked with Cu^2+^ was compared with reference values obtained from ICP-MS, presented in Table S2. Bottled purified water samples, which were considered to contain fewer impurities, exhibited the best recovery results both in the case of low and high Cu^2+^ concentrations. Moreover, due to the presence of various mineral components, the detection results obtained from the lake water sample were slightly increased compared with bottled purified water samples. In contrast, the detection results of the water sample from domestic sewage were considerably increased compared with the spiked amount of the Cu^2+^ standard mainly due to the inherent Cu^2+^ residual in domestic sewage. In general, the detection results were similar between our strategy and ICP-MS in samples at different Cu^2+^ concentrations and the developed Cu^2+^ turn-off sensor exhibited satisfactory accuracy for Cu^2+^ detection in real water samples.

## Conclusion

In conclusion, we constructed a rapid and visual turn-off sensor for Cu^2+^ detection, displaying several excellent features: (1) *Turn*-*off* type. The detection unit was composed of HCR-based HRP concatemers cross-linked with the substrate strand of Cu^2+^-dependent DNAzyme via Watson-Crick base pairing. Upon Cu^2+^ addition, Cu^2+^-dependent DNAzyme was irreversibly cleaved, bringing about dramatic decline of the signal output. Thus, the turn-off sensor could effectively get over false positives that interfere with the results. (2) *Visualization*. As the signal amplifier and signal report element, HCR-based HRP concatemers could catalyze hydrogen peroxide (H_2_O_2_) via TMB, generating obvious green color and turning yellow after sulfuric acid termination with optical absorption at 450 nm. The signal output could be semi-quantitatively observed by the naked eye with the minimum target of 500 nM and quantitatively monitored by a portable spectrophotometer with the LOD of 8 nM, demonstrating immense potential for on-site detection. (3) *Sensitivity, Selectivity* and *anti*-*interference*. The turn-off sensor possesses outstanding sensitivity, selectivity and anti-interference, exhibiting a linear calibration from 0.05 to 3 μM with a detection limit of 8 nM and displaying remarkable applicability for monitoring Cu^2+^ in real water samples, by virtue of the specificity of DNAzyme and the signal amplification of HCR-based HRP concatemers. (4) *Rapidity*. Given that the immobilized Cu^2+^-dependent DNAzyme coupled with HCR-based HRP concatemers was prepared on the surface in advance, the actual reaction time after the Cu^2+^ sample introduction was 5 min, indicating great promise for on-site rapid testing.

## Additional Information

**How to cite this article:** Xu, W. *et al*. A rapid and visual turn-off sensor for detecting copper (II) ion based on DNAzyme coupled with HCR-based HRP concatemers. *Sci. Rep.* **7**, 43362; doi: 10.1038/srep43362 (2017).

**Publisher's note:** Springer Nature remains neutral with regard to jurisdictional claims in published maps and institutional affiliations.

## Supplementary Material

Supporting Information
