# Supplementary material for: A rapid and visual turn-off sensor for detecting copper (II) ion based on DNAzyme coupled with HCR-based HRP concatemers
Source: Sci Rep. 2017 Mar 7;7:43362. doi: 10.1038/srep43362 (PMC5339725; doi:10.1038/srep43362)
Supplement: Supporting Information [file srep43362-s1.pdf]

Supporting Information

A rapid and visual turn-off sensor for detecting copper (II) ion based on DNAzyme coupled with HCR-based HRP concatemers

Wentao Xu[1, 2], Jingjing Tian[2], Yunbo Luo[1], Longjiao Zhu[2], Kunlun Huang[1,2 *]

[1]Beijing Advanced Innovation Center for Food Nutrition and Human Health, College of Food Science & Nutritional Engineering, China Agricultural University, Beijing 100083, China

[2]Beijing Laboratory for Food Quality and Safety, College of Food Science and Nutritional Engineering, China Agricultural University, Beijing 100083, China

[*] To whom correspondence should be addressed.

Figure S1 Chromogenic results based on HCR-based HRP complex

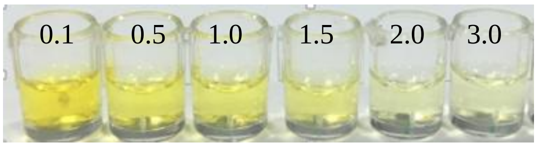

Figure S1 chromogenic results of the Cu^{2+} turn-off sensor with different concentrations of Cu^{2+} from 0.1 to 3.0 μM.

Table S1 Detection Limits of Cu^{2+} Colorimetric Determination

LOD	Research group
1 μM	Zhang et al. 2012[1]
4 nM	Liu et al. 2011[2]
5 nM	Shimron et al. 2011[3]
20 nM	Liu and Lu 2007[4]
250 nM	YunáZhang and JamesáYang 2011[5]
20 μM	Jo et al. 2012[6]
3 μM	Qu et al. 2011[7]
100 nM	Ge et al. 2014[8]

Table S2 Determination of Cu^{2+} in three different water samples using the Cu^{2+} turn-off sensor and ICP-MS (n=3).

Sample	Cu^{2+} (μM) spiked	Cu^{2+} turn-off sensor		ICP-MS	
		Detected mean [a] ± SD [b]	Recovery (%)	Detected mean [a] ± SD [b]	Recovery (%)
Bottled purified Water	0.500	0.482±0.061	96.4	0.491±0.037	98.2
	2.500	2.397±0.920	95.9	2.321±0.029	92.8
Lake water	0.500	0.544±0.047	100.1	0.523±0.043	104.6
	2.500	2.682±0.028	107.3	2.734±0.052	109.4
Domestic sewage sample	0.500	1.227±0.035	245.4	1.155±0.039	231.0
	1.500	2.768±0.041	184.5	2.814±0.211	187.6

[a] Mean value of three individual determinations.

[b] Standard deviation.

REFERENCES

1 Zhang, Q., Cai, Y., Li, H., Kong, D.-M. & Shen, H.-X. Sensitive dual DNAzymes-based sensors designed by grafting self-blocked G-quadruplex DNAzymes to the substrates of metal ion-triggered DNA/RNA-cleaving DNAzymes. *Biosensors and Bioelectronics* **38**, 331-336 (2012).

2 Liu, M. *et al.* A “turn-on” fluorescent copper biosensor based on DNA cleavage-dependent graphene-quenched DNAzyme. *Biosensors and Bioelectronics* **26**, 4111-4116 (2011).

3 Shimron, S., Wang, F., Orbach, R. & Willner, I. Amplified detection of DNA through the enzyme-free autonomous assembly of hemin/G-quadruplex DNAzyme nanowires. *Analytical chemistry* **84**, 1042-1048 (2011).

4 Liu, J. & Lu, Y. Colorimetric Cu^{2+} detection with a ligation DNAzyme and nanoparticles. *Chemical Communications*, 4872-4874 (2007).

5 Yun âZhang, W. & James âYang, C. DNAzyme crosslinked hydrogel: a new platform for visual detection of metal ions. *Chemical Communications* **47**, 9312-9314 (2011).

6 Jo, J. *et al.* Reactivity-based detection of copper (II) ion in water: oxidative cyclization of azoaromatics as fluorescence turn-on signaling mechanism. *Journal of the American Chemical Society* **134**, 16000-16007 (2012).

7 Qu, W., Liu, Y., Liu, D., Wang, Z. & Jiang, X. Copper ‐ Mediated Amplification Allows Readout of Immunoassays by the Naked Eye. *Angewandte Chemie International Edition* **50**, 3442-3445 (2011).

8 Ge, C. *et al.* Colorimetric detection of copper (II) ion using click chemistry and Hemin/G-quadruplex horseradish peroxidase-mimicking DNAzyme. *Analytical chemistry* **86**, 6387-6392 (2014).
